# A decellularized and sterilized human meniscus allograft for off-the-shelf meniscus replacement

**DOI:** 10.1186/s40634-022-00555-y

**Published:** 2022-12-05

**Authors:** Janne Spierings, Wietske Velthuijs, Amal Mansoor, Manon E. Bertrand, Jorge Alfredo Uquillas, Keita Ito, Rob P. A. Janssen, Jasper Foolen

**Affiliations:** 1 Orthopaedic Biomechanics, Department of Biomedical Engineering, Eindhoven University of Technology, PO Box 513, 5600 MB Eindhoven, the Netherlands; 2 Institute of Complex Molecular Systems, Eindhoven University of Technology, Eindhoven, The Netherlands; 3 Hightech Contract Manufacturing Medical, Nijmegen, The Netherlands; 4 Maxima Medical Centre Eindhoven/Veldhoven, Eindhoven, The Netherlands; 5 Health Innovations and Technology, Department of Paramedical Sciences, Fontys University of Applied Sciences, Eindhoven, The Netherlands

**Keywords:** Meniscus, Meniscal reconstruction, Allograft, Decellularization, Supercritical carbon dioxide sterilization

## Abstract

**Purpose:**

Meniscus tears are one of the most frequent orthopedic knee injuries, which are currently often treated performing meniscectomy. Clinical concerns comprise progressive degeneration of the meniscus tissue, a change in knee biomechanics, and an early onset of osteoarthritis. To overcome these problems, meniscal transplant surgery can be performed. However, adequate meniscal replacements remain to be a great challenge. In this research, we propose the use of a decellularized and sterilized human meniscus allograft as meniscal replacement.

**Methods:**

Human menisci were subjected to a decellularization protocol combined with sterilization using supercritical carbon dioxide (scCO_2_). The decellularization efficiency of human meniscus tissue was evaluated via DNA quantification and Hematoxylin & Eosin (H&E) and DAPI staining. The mechanical properties of native, decellularized, and decellularized + sterilized meniscus tissue were evaluated, and its composition was determined via collagen and glycosaminoglycan (GAG) quantification, and a collagen and GAG stain. Additionally, cytocompatibility was determined in vitro.

**Results:**

Human menisci were decellularized to DNA levels of ~ 20 ng/mg of tissue dry weight. The mechanical properties and composition of human meniscus were not significantly affected by decellularization and sterilization. Histologically, the decellularized and sterilized meniscus tissue had maintained its collagen and glycosaminoglycan structure and distribution. Besides, the processed tissues were not cytotoxic to seeded human dermal fibroblasts in vitro.

**Conclusions:**

Human meniscus tissue was successfully decellularized, while maintaining biomechanical, structural, and compositional properties, without signs of in vitro cytotoxicity. The ease at which human meniscus tissue can be efficiently decellularized, while maintaining its native properties, paves the way towards clinical use.

**Supplementary Information:**

The online version contains supplementary material available at 10.1186/s40634-022-00555-y.

## Background

With over 850,000 surgical interventions each year in the United States alone, meniscus tears are among the most frequent orthopedic knee injuries [1]. They are caused by acute sports-related injuries in young people or by long-term degeneration in older patients and result in pain, reduced productivity, and decreased quality of life [23, 27, 39]. Due to the dense composition, low cellularity, and variable vascularity of the meniscus tissue, its self-healing capacity is limited [10, 18].

When a meniscus is (partially) injured or degenerated beyond repair, a (partial) meniscectomy is performed [21], which leads to a change in load-bearing capacity of the meniscus. This often results in degenerative changes, altered knee biomechanics, and an early onset of osteoarthritis [36, 39]. Therefore, eventually meniscal transplant surgery can be performed. However, adequate meniscal replacements remain to be a great challenge. Numerous synthetic and biological materials have been used, with collagen-based and polyurethane-based meniscus scaffolds being already available in clinics [38, 43]. Despite good mid-term clinical outcomes, it remains questionable whether these treatments can provide a long-term chondroprotective effect and an improvement of long-term clinical outcomes [6, 9, 14, 20, 35]. Alternatively, allografts can be used in meniscal reconstruction. They have a similar structure as native menisci but can cause host reactions leading to subsequent failure of the graft and are at risk of disease transmission [4, 41].

These complications can be diminished by using decellularized allografts. Decellularization is the process of removing allogeneic cellular components, thus reducing the risk of disease transmission, immune rejection and inflammation while maintaining the integrity of the extracellular matrix (ECM) [3, 32]. For adequate decellularization of tissues for re-implantation, the Badylak lab proposed three requirements: after decellularization, the graft should have 1) less than 50 ng of double-stranded DNA per mg dry weight; 2) no visible nuclear material, and 3) DNA fragments smaller than 200 base pairs in length [3]. Compared to fresh allografts and biological or synthetic scaffolds, porcine decellularized meniscus tissue already showed advantageous outcomes regarding retention of major structural proteins and histoarchitecture and in vitro cell survival and proliferation [15, 34]. Additionally, another study showed that decellularization of bovine meniscus tissue resulted in a loss of sulfated glycosaminoglycans (GAGs) and minor disruptions in collagen structure [42]. Still, decellularized menisci are immunologically superior to freeze-dried menisci, as shown in an in vivo rabbit model [15], attract less inflammatory cells than native tissue and prevent severe host reactions [12, 15]. Thus, decellularized allografts have been shown to have great potential, but there is room for improvement.

To reduce the risk of disease transmission and ensure safety, meniscal allografts need to be sterilized [2, 26]. The most common method, gamma irradiation, has been shown to adversely affect tissue structure and biomechanical properties [2, 26]. Contrarily, newer methods such as supercritical carbon dioxide (scCO_2_) sterilization have been used to sterilize ovine menisci while preserving native tissue structure and mechanical properties [2].

To build on this, we aim to produce a decellularized and sterilized human meniscus allograft without affecting its important biological and mechanical properties. We thus asked whether we could exploit our previously developed decellularization/sterilization protocol to decellularize and sterilize human meniscus tissue without significantly affecting its properties. Results of this study will contribute to develop in vivo approaches towards an off-the-shelf product for total meniscus replacement.

## Materials and methods

### Tissue harvest and storage

Nine pairs of human cadaveric legs (age = 82 ± 10 years old, 5 male, 4 female) were obtained from the Radboud University Medical Center (Department of Medical Imaging, Anatomy, Radboudumc, Nijmegen, The Netherlands). All procedures regarding the use of human tissue were strictly obeyed Article 67–69, of the ‘Wet op de Lijkbezorging’. None of the cadaveric legs had previous operative treatments of the knee joint. Specimens were wrapped in saline solution-soaked gauze and stored in a −20 °C freezer upon arrival. Legs were thawed at room temperature for 24 hours before tissue procurement. Menisci were procured by an incision perpendicular to the meniscal horn attachments and human remains were transported back (after harvesting of other tissues, e.g. tendons, for other research purposes) to the Radboud University Medical Center for anonymous cremation. After procurement, menisci were washed in phosphate buffered saline (PBS) for 1 hour at room temperature to remove excess blood and stored at −20 °C until further processing. Only macroscopically intact menisci without fibrillations or ossifications were used. In total, 14 medial (width = 46.8 ± 6.3 mm, thickness = 5.2 ± 0.7 mm) and 14 lateral menisci (width = 42.0 ± 2.7 mm, thickness = 6.7 ± 1.0 mm) were included in this study.

### Decellularization

Decellularization was carried out based on a developed protocol previously developed for tendons and ligaments [5, 37]. In short, frozen menisci tissue was freeze-thawed five times in liquid nitrogen and 37 °C distilled water, washed in distilled water at room temperature overnight, and soaked in 1% Triton X-100 (Merck) in a Tris-EDTA buffer (pH 7.6; Merck, Sigma-Aldrich) for 24 hours. Then, samples were shortly washed in distilled water before soaking them for 24 hours in Benzonase (25 U/ml; Sigma-Aldrich) in a Tris buffer containing magnesium chloride hexahydrate (37 °C, pH 7.6; Sigma-Aldrich). Samples were then subjected to three washes of one hour in PBS + 2.7 mM EDTA (pH 7.6) at 37 °C and overnight incubation in distilled water at 4 °C. Next, samples were soaked in 1% Triton X-100 for 72 hours, shortly washed in distilled water, soaked in Benzonase for 48 hours at 37 °C, and washed three times 1 hour each in PBS + 2.7 mM EDTA at 37 °C. Finally, samples were washed in distilled water for 2 days. Distilled water was replaced every day. All steps were performed at room temperature on a rotator shaker unless stated otherwise. After the decellularization procedure, samples were stored at −80 °C until further usage.

### Sterilization

Sterilization was performed in a Supercritical Fluid Extractor system (Waters Corporation) using supercritical carbon dioxide and peracetic acid (HCM Medical, Nijmegen). The sterilization procedure consisted of three steps: 1) A static process for 15 min with a flow of 20 g/min, a vessel temperature of 25 °C, and a pressure of 60 bar. 2) A second static process for 240 min with a flow of 20 g/min, a vessel temperature of 37 °C, and a pressure of 160 bar. 3) A dynamic flushing step, at a flow of 30 g/min, a vessel temperature of 37 °C, and a pressure of 160 bar. Samples were stored at −20 °C until further usage.

### Sample digestion

To measure DNA, GAGs, and hydroxyproline (HYP), samples were digested, according to Kim et al. [19]. Briefly, a small piece (~ 3 mm × 3 mm) from the middle portion of each sample was taken and lyophilized for 48 hours. Three pieces of 2–3 mg of freeze-dried tissue were used as technical replicate from each meniscus and digested in 500 μL Papain digestion buffer [130 μg Papain (Sigma-Aldrich) per ml in 5 mM L-cysteine hydrochloride (Sigma-Aldrich) + 5 mM Na_2_EDTA (VWR)] for 24 hours at 60 °C in an Eppendorf ThermoMixer® at 300 rpm.

### Determination of DNA content

The double-stranded DNA (dsDNA) content was quantified in decellularized (*N* = 8), decellularized + sterilized (*N* = 8), and native (2x *N* = 8) samples using the Qubit dsDNA High Sensitivity assay (Thermo Fisher Scientific) according to the manufacturers’ protocol. Readings were taken using the Qubit® 2.0 Fluorometer and data was normalized to tissue dry weight, obtained before digestion.

### Visualization of cell nuclei

#### Preparation of tissue sections

Tissue sections of native (*N* = 3), decellularized (*N* = 3), and decellularized + sterilized (*N* = 3) samples were embedded in OCT Compound (Tissue-Tek®) and frozen on dry ice. Embedded samples were sectioned longitudinally at 6 μm and were fixed in 3.7% formaldehyde (Merck) for 20 min.

#### Hematoxylin and eosin stain

To visualize the remaining nuclei after decellularization and sterilization, tissue sections were stained with Hematoxylin and Eosin (H&E). Sections were incubated in Mayer’s Hematoxylin solution (Sigma-Aldrich) for 10 min, washed in distilled and tap water, incubated in aqueous Eosin Y solution (Sigma-Aldrich) for 1 min, and washed again. Finally, tissue sections were dehydrated in increasing series of ethanol and mounted with Entellan (Merck).

#### DAPI stain

To identify remaining cell nuclei after decellularization and sterilization, tissue sections were stained with 4′,6-diamidino-2-phenylindole dihydrochloride (DAPI; Sigma-Aldrich). Tissue sections were incubated in 1 μg/mL DAPI solution for 30 min and mounted with Mowiol (Merck).

### Mechanical testing

**Fig. 1:**
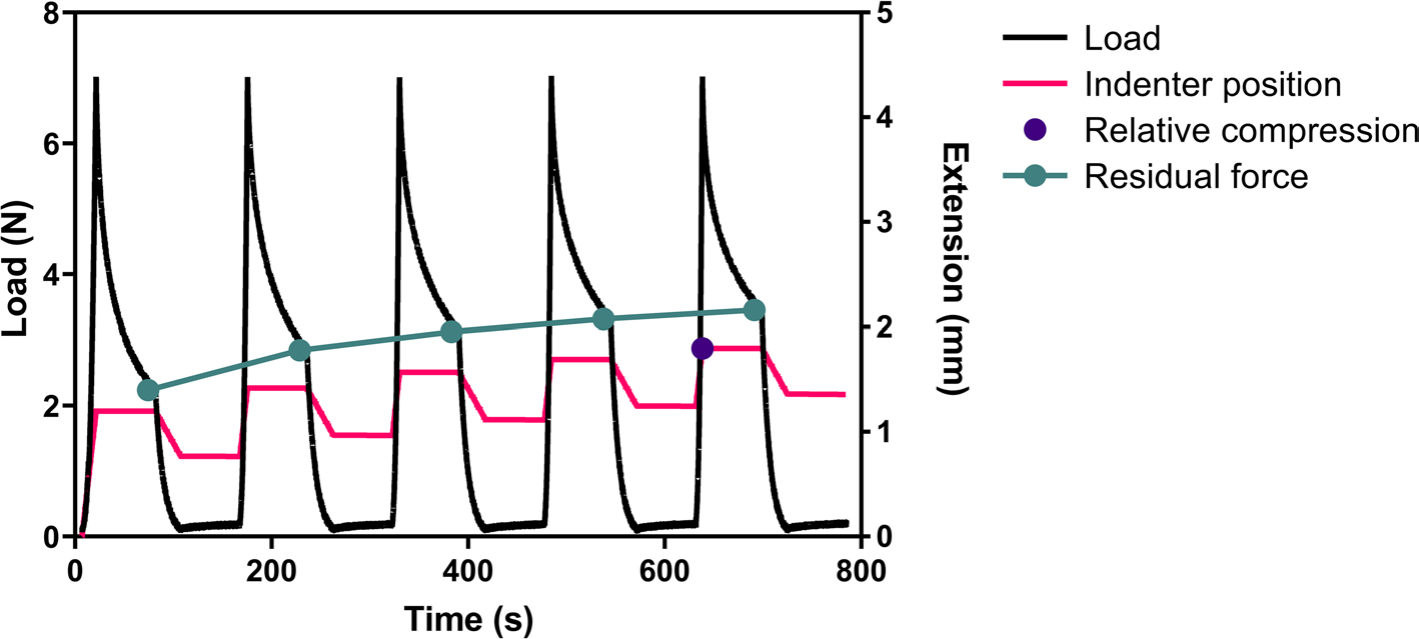
Graphical overview showing a typical load curve of the mechanical test consisting of five test cycles. The graphical course of preload, compression, relaxation, and unloading is shown in black. Relative compression (purple dot) was calculated from the indenter position at the end of dynamic compression of the 5th test cycle. The residual force was defined as the recorded load at the end of static compression of each test cycle. Load and residual force are shown on the left y-axis and indenter position and relative compression on the right y-axis

Mechanical properties of medial menisci samples of five donors were determined using an unconfined stress-relaxation ball indentation test. Samples were prepared according to a previously described protocol by Maier et al. [24]. In short, one cylinder (diameter = 6 mm) per meniscus sample was punched out of the central portion using a biopsy punch (kai Europe GmbH). The superior and inferior surface of the cylindrical samples were cut parallel, resulting in cylinders of about 3 mm in height. During testing, the samples were placed in a custom-designed mold with a circular cavity (diameter = 6 mm, depth = 0.3 mm) to prevent lateral movement of the samples during testing. The custom-made indenter and tip consisted of a steel ball (diameter = 6 mm). Mechanical testing was performed on a mechanical tester (Criterion Model 42, MTS, Eden Prairie, Minnesota) with a 50 N load cell. A test cycle consisted of the following four phases: 1) Preloading at 0.1 N; 2) Dynamic compression from pre-load to 7 N at a velocity of 5 mm/min; 3) Stress relaxation at 7 N for 1 min (the indenter position was fixed at 7 N), and 4) Unloading to 0.15 N at a velocity of 1 mm/min. After an interval of 1 min, the next test cycle started, with a total of five repetitions. Load, indenter position, and time were recorded during testing. A graphical overview of one test cycle can be found in Fig. 1.

From the data, relative compression, as an indicator for tissue viscosity, was calculated from the indenter position at the end of dynamic compression in the 5th test cycle. The Young’s modulus was determined during the 2nd step of each test cycle, calculated from the linear slope of the engineering stress-strain curve. And finally, the residual force, a measure of the tissue’s viscoelastic behavior, was defined as the recorded load at the end of the 3rd step (static compression) of each test cycle.

### Determination of hydroxyproline and glycosaminoglycan content

The total HYP content of decellularized (*N* = 8), decellularized + sterilized (*N* = 8), and native (2x *N* = 8) meniscus was determined, as a measure of collagen, according to Huszar et al. [16]. Total sulfated GAG content was quantified using a dimethyl methylene blue (DMMB) assay according to Farndale et al. [8]. A more detailed description of these assays can be found in the Supplements.

### Structural properties

#### Picro Sirius red stain

The effect of decellularization and/or sterilization on collagen fibers was visualized using the Picro Sirius Red Stain Kit (Connective Tissue Stain; Abcam). Hydrated tissue sections were incubated in Picro Sirius Red solution for 60 min and rinsed with 3% acetic acid and absolute ethanol. Then, tissue sections were dehydrated in absolute ethanol and mounted with Entellan.

#### Alcian blue stain

Alcian Blue was used to visualize the effect of decellularization and/or sterilization on glycosaminoglycans. In short, tissue sections were incubated in 3% acetic acid solution (Abcam) for 3 min, incubated in 1% Alcian Blue solution (pH 2.5; Alcian Blue-8GX (Sigma Aldrich) dissolved in 3% Acetic Acid) for 30 min, and washed in 3% acetic acid, tap water, and distilled water. Subsequently, tissue sections were incubated in Nuclear Fast Red solution (Abcam) for 5 min, and washed again. Tissue sections were dehydrated in increasing series of ethanol and mounted with Entellan.

### Determination of cell cytotoxicity

Tissue sections of native (*N* = 3), decellularized (*N* = 3), and decellularized + sterilized (*N* = 3) samples were embedded in OCT Compound and frozen on dry ice. Then, samples were longitudinally sectioned at 300 μm thickness. Meniscus sections were freeze-dried for 48 hours, punched to a diameter of 4 mm, in order to fit the well, and sterilized in serial dilutions of ethanol, 70%, 80%, 90%, and absolute, for 20 min each, followed by UV sterilization for 15 min. Once sterilized, the sections were washed in sterile PBS three times 20 min each and once in culture media (DMEM [4.5 g/mL glucose, 4.0 mM L-glutamine, 25 mM HEPES], 10% FBS, 1% P/S, 1% nonessential amino acids) for 20 min. Finally, tissue sections were incubated in culture media overnight at 37 °C and 5% CO_2_.

Human dermal fibroblasts (NHDF-Ad, Lonza CC-2511) at passage 7 were seeded on the meniscus tissue sections (2000 cells per tissue) and submerged in 100 μL culture media and incubated at 37 °C and 5% CO_2_. To determine cell cytotoxicity, an assay measuring Lactate Dehydrogenase (LDH) release was performed using the CyQUANT Cytotoxicity Assay Kit (Invitrogen) according to manufacturer’s protocol. Experiments were performed on day 1, 4, and 7. Cells cultured on a flat polystyrene surface were used as a negative control (0% cytotoxicity) and the maximum LDH release as a positive control (100% cytotoxicity).

### Statistical analysis

Since there are structural differences between the medial and lateral meniscus, the meniscus from left or right knee, and within the meniscus itself [25], it was chosen to perform the statistical analysis in an unpaired fashion. All statistical analyses were performed in GraphPad Prism version 9.1 (GraphPad Software, Inc., San Diego, CA). One-way ANOVA or Kruskal-Wallis tests with, respectively, Šídák or Dunn’s post hoc multiple comparison tests were performed with significance level set at *p* < 0.05. Results are expressed as mean ± standard deviation.

## Results

### Decellularization efficiency

**Fig. 2:**
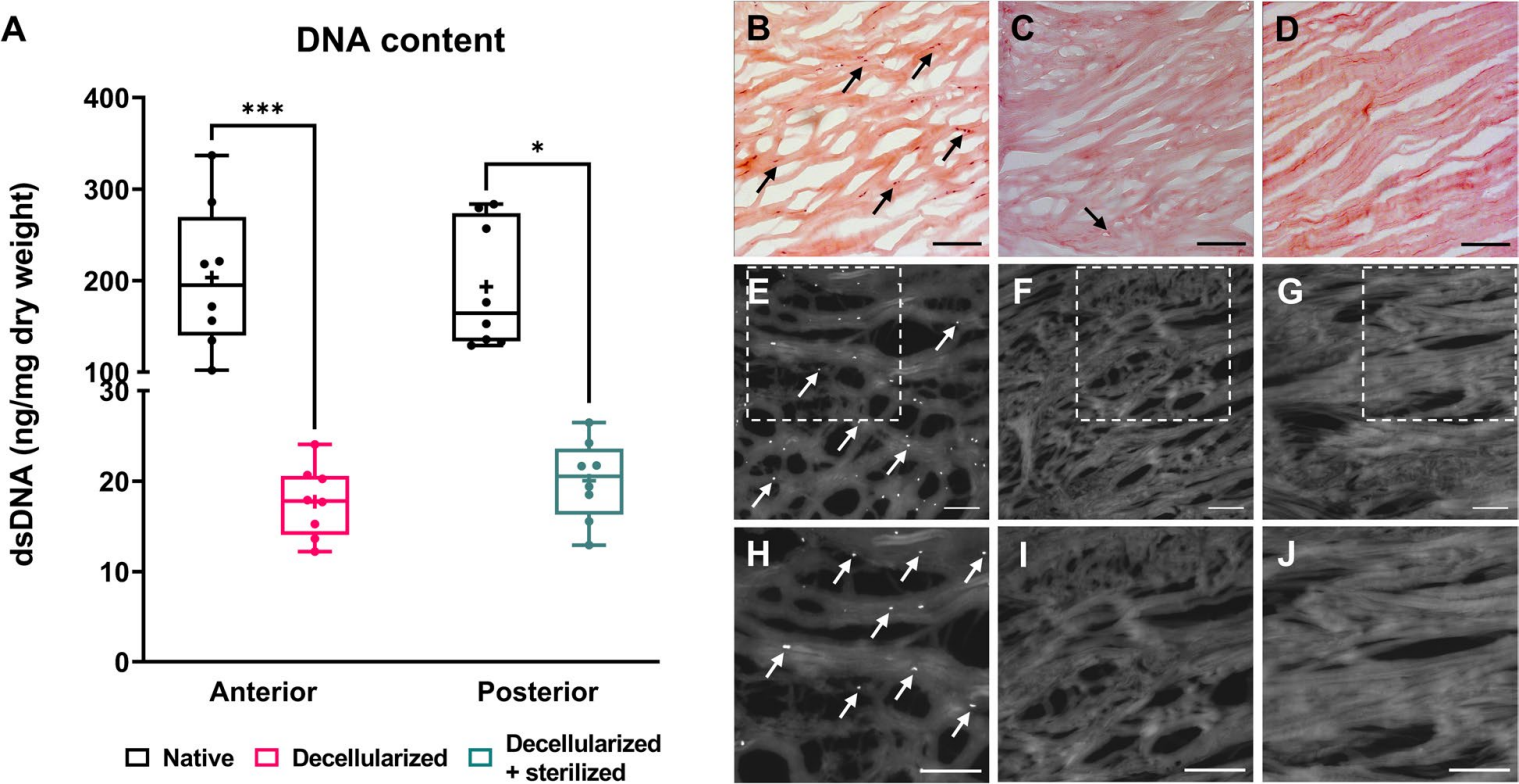
Meniscus tissue is efficiently decellularized. **A** DNA content in native, decellularized and decellularized + sterilized meniscus tissue. * indicates a *p*-value of < 0.05, *** indicates a p-value of < 0.001. Box plots show all data points with median, maximum value, minimum value, and 25th and 75th percentiles. **B**-**D** Histological sections of native (**B**), decellularized (**C**), and decellularized + sterilized meniscus (**D**) were stained with H&E. **E**-**G** Histological sections of native (**E**), decellularized (**F**), and decellularized + sterilized meniscus (**G**) were stained with DAPI. **H**-**I** Higher magnification of DAPI stained sections of native (**H**), decellularized (**I**), and decellularized + sterilized meniscus (**J**). Cell nuclei are indicated by arrows. Scale bar represents 100 μm

To evaluate the efficiency of decellularization and/or sterilization, remaining dsDNA content was quantified. Decellularized meniscus tissue contained 17.7 ± 3.7 ng DNA per mg tissue dry weight, which is significantly lower than the native contralateral control with 203.3 ± 73.7 ng/mg tissue dry weight (Kruskal-Wallis, *p* < 0.001). The remaining DNA content in decellularized and sterilized meniscus tissue was 20.1 ± 4.2 ng DNA per mg tissue dry weight, also significantly lower than the native contralateral control, which contained 193.3 ± 63.8 ng DNA per mg tissue dry weight (Kruskal-Wallis, *p* < 0.05) (Fig. 2A). In both decellularized and decellularized + sterilized meniscus tissue, the remaining DNA content is lower than the threshold set by the decellularization criteria of the Badylak lab [13].

Nuclei were stained using H&E and DAPI staining. On both H&E and DAPI stained native tissue sections, cell nuclei (indicated by arrows) were observed (Fig. 2B, E, H). The cells were aligned between the collagen fibers of the ECM. After decellularization, sparse cell nuclei could still be observed (Fig. 2C), however after subsequent sterilization cell nuclei were absent (Fig. 2D, G, J).

### Mechanical properties are maintained after decellularization and sterilization

The viscoelastic behavior of meniscus tissue was assessed using an unconfined indentation stress relaxation test over several cycles. All samples were loaded to 7 N without signs of plastic deformation and the slopes of the load curves were found to be linear between 2 N and 5 N (Fig. 1).

**Fig. 3:**
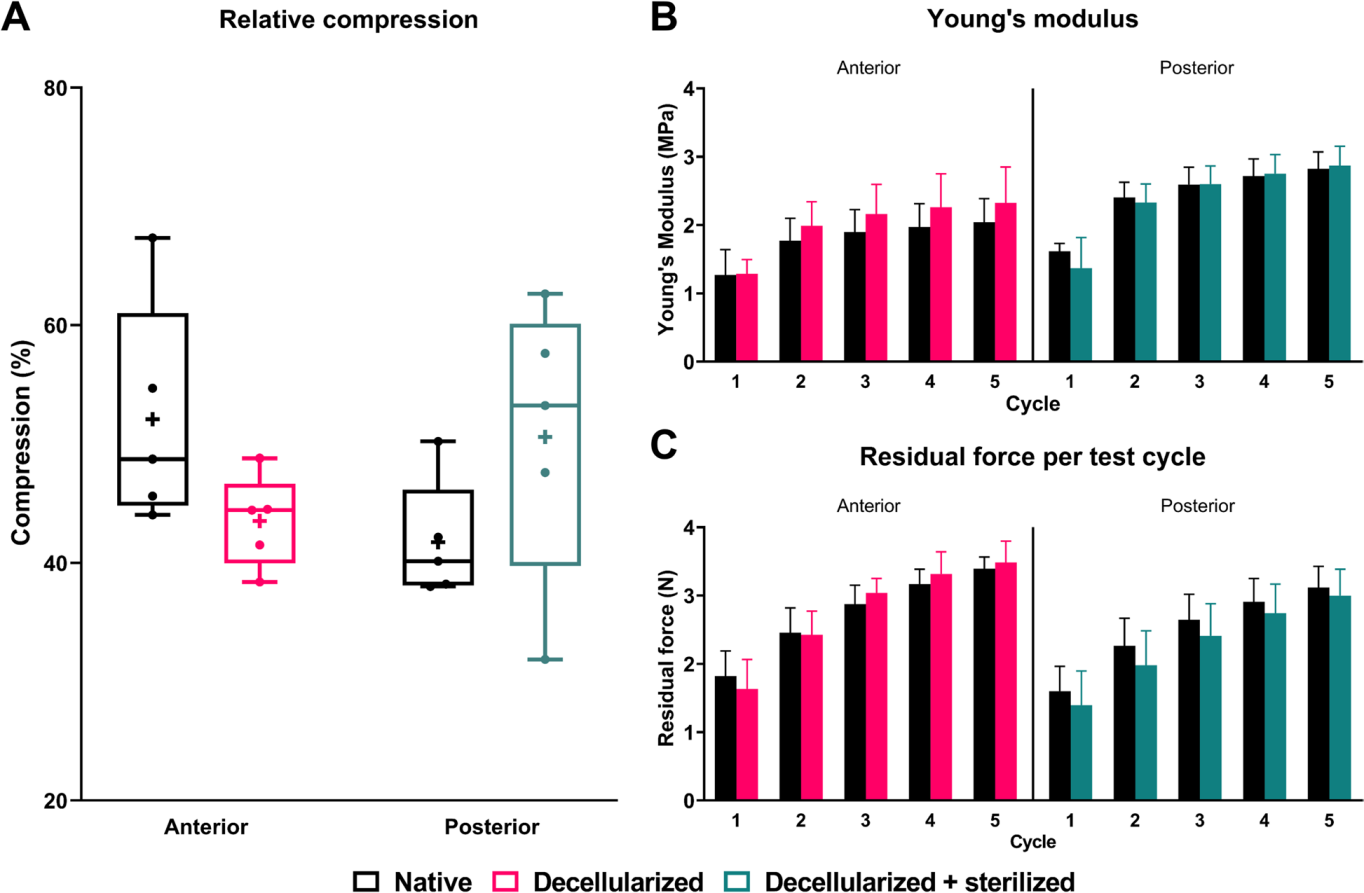
Native viscoelastic behavior of human meniscus tissue is maintained after both decellularization and/or sterilization. **A** Percentual change in compression after test cycle 5 compared to relative sample height. **B** Young’s modulus per test cycle. **C** Residual force per test cycle

The relative compression was found to be 44 ± 3% in decellularized samples and 51 ± 11% in decellularized and sterilized samples. Both were not significantly different from the native contralateral control (52 ± 8% (one-way ANOVA, *p* = 0.40) and 42 ± 4% (one-way ANOVA, *p* = 0.37), respectively) (Fig. 3A).

Next, for the Young’s modulus, there were no significant differences found between the decellularized and native samples (Kruskal-Wallis, *p* > 0.99) and between the decellularized + sterilized and native counterparts (Kruskal-Wallis, *p* > 0.99), in each individual test cycle (Fig. 3B). Similarly, the residual force in each individual test cycle, was not significant different between the different groups (Kruskal-Wallis, *p* > 0.99) (Fig. 3C). For both the stiffness and the residual force, a time dependent increase was observed.

Together, these results indicate that the mechanical properties of native meniscal tissue are maintained after decellularization and/or sterilization.

### Biochemical composition and tissue structure are maintained after decellularization and sterilization

**Fig. 4:**
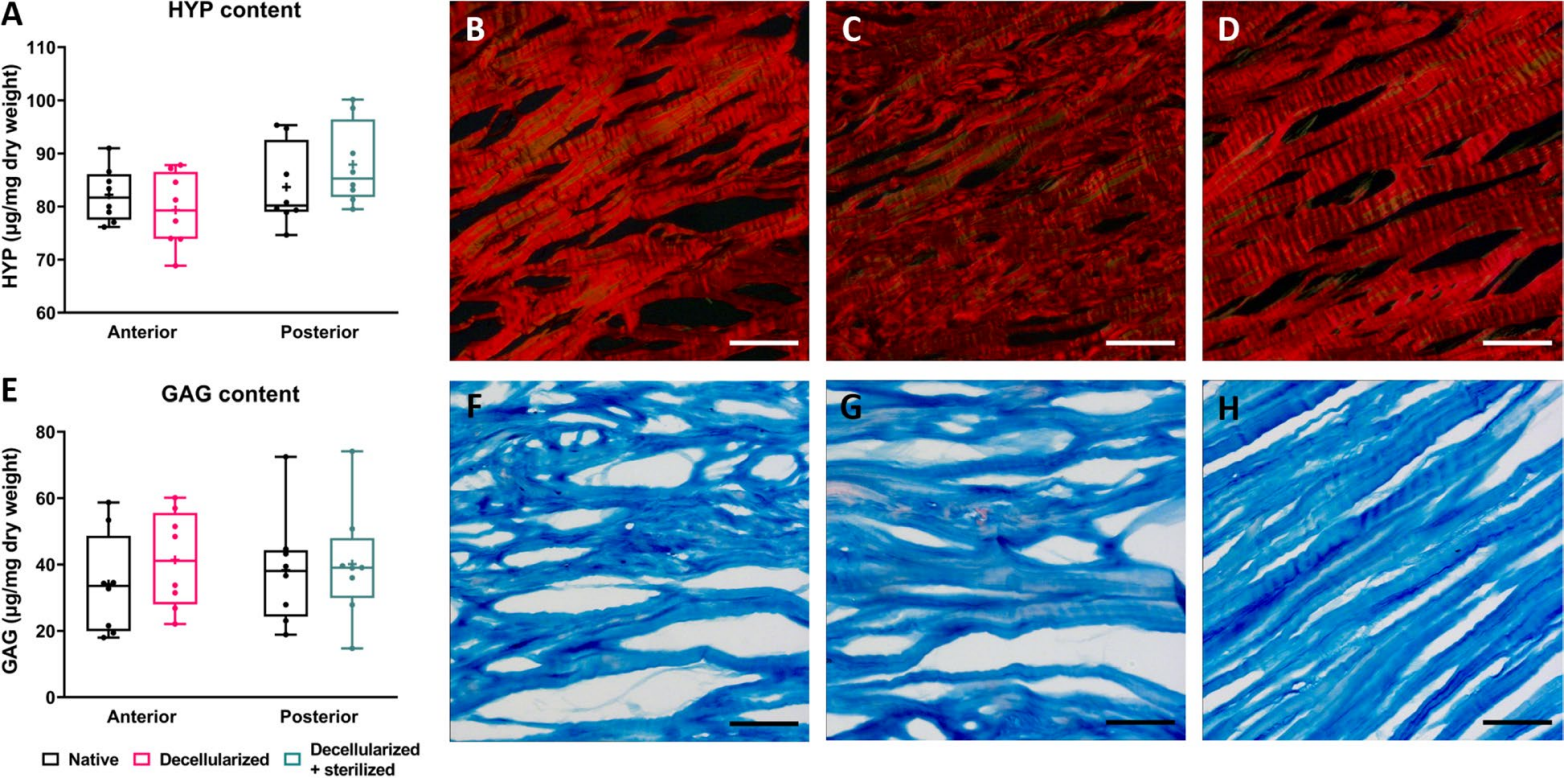
Native meniscal biochemical composition and tissue structure are maintained after both decellularization and sterilization. **A** Hydroxyproline content. **B**-**D** Histological sections of native (**B**), decellularized (**C**), and decellularized + sterilized meniscus (**C**) were stained with Picro Sirius Red. **E** Glycosaminoglycan content. **F**-**H** Histological sections of native (**F**), decellularized (**G**), and decellularized + sterilized meniscus (**H**) were stained with Alcian Blue. Box plots show all data points with median, maximum value, minimum value, and 25th and 75th percentiles. Scale bar represents 100 μm

To evaluate the retention of collagen after decellularization and sterilization, hydroxyproline content in native, decellularized, and decellularized + sterilized meniscus samples was quantified. The total HYP content in decellularized samples was 79.4 ± 6.5 μg per mg tissue dry weight. This was not significantly different compared to the native contralateral control, which contained 82.2 ± 4.8 μg hydroxyproline per mg tissue dry weight (one-way ANOVA, *p* = 0.88). Decellularized + sterilized samples were composed of 87.9 ± 7.7 μg hydroxyproline per mg tissue dry weight, which was not significantly different from native contralateral controls with 83.7 ± 7.2 μg/mg dry weight (one-way ANOVA, *p* = 0.65) (Fig. 4A). This indicates that decellularization and/or sterilization did not significantly affect the hydroxyproline content.

Next to hydroxyproline content, collagen structure was also evaluated. Using Picro Sirius Red, the collagen distribution within the tissue was visualized. The typical crimp pattern of the collagen fibers was observed for all groups. No gross evidence of fiber damage was observed (Fig. 4B-D).

In decellularized meniscus tissue, GAG content was found to be 41.4 ± 13.6 μg/mg tissue dry weight, against 34.1 ± 14.2 μg/mg tissue dry weight in the native contralateral control. Decellularized + sterilized samples had a GAG content of 38.3 ± 15.6 μg/mg tissue dry weight. The contralateral control contained 40.1 ± 16.1 μg GAGs per mg tissue dry weight. There were no significant differences found between groups (one-way ANOVA, *p* = 0.84 and *p* = 0.99, respectively) (Fig. 4E).

On Alcian Blue stained tissue sections, a homogenous GAG distribution was found in all groups. There were no differences observed in GAGs between native, decellularized, or decellularized and sterilized stained tissue sections (Fig. 4F-H).

Taking these results together, both compositional and structural properties of native meniscus tissue were maintained after decellularization and/or sterilization.

### Decellularized and sterilized meniscus sections are not cytotoxic in vitro

**Fig. 5:**
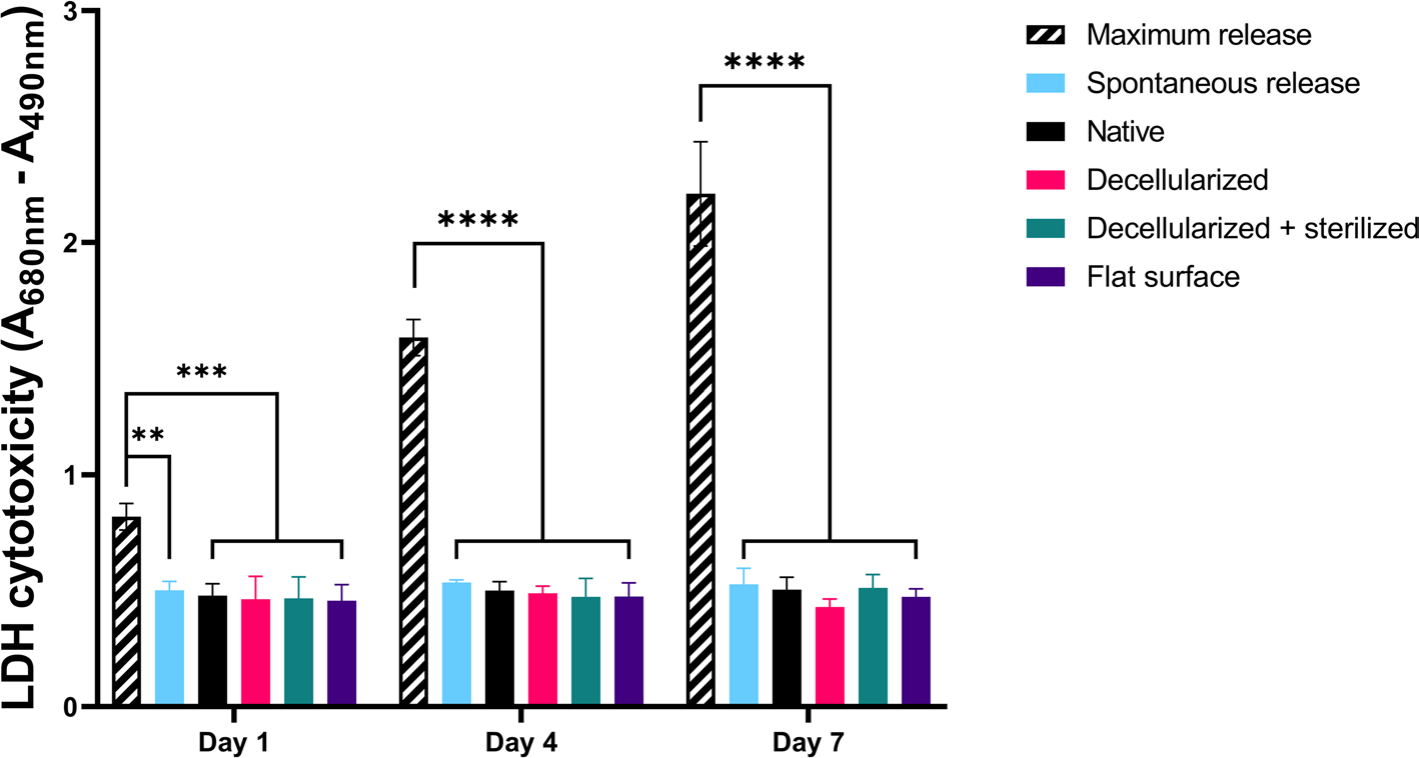
Human decellularized and/or sterilized meniscus tissue is not cytotoxic. LDH cytotoxicity is measured as the LDH release by human dermal fibroblasts when seeded on native meniscus, decellularized meniscus, decellularized + sterilized meniscus, or flat polystyrene. ** indicates a *p*-value of < 0.01, *** indicates a *p*-value of < 0.001, **** indicates a *p*-value of < 0.0001

The LDH release of human dermal fibroblasts on human native, decellularized, and decellularized + sterilized meniscus tissue sections was quantified (Fig. 5). There were no significant differences found between native, decellularized, and decellularized + sterilized human meniscus tissue sections (one-way ANOVA, *p* > 0.92) and the LDH cytotoxicity was found to be equal to the spontaneous LDH release of the cells (one-way ANOVA, *p* > 0.76). Besides, the LDH release of human dermal fibroblast seeded on a standard flat polystyrene surface was not significantly different from that of the experimental groups (one-way ANOVA, *p* > 0.99). In addition, the LDH release was constant during 7 days of in vitro culture. The maximum LDH release increased over time (one-way ANOVA, *p* < 0.0001), indicative for an increase in total cell number (proliferation).

## Discussion

Current strategies for meniscus replacement can be further optimized to provide a long-term chondroprotective effect and thus clinical outcome. For this reason, this study aimed to create a decellularized and sterilized human meniscus allograft while maintaining its mechanical, compositional, and structural properties. Results demonstrated that human meniscal tissue could be decellularized to meet previously set decellularization criteria (dsDNA < 50 ng/mg tissue dry weight, and absence of visible cell nuclei on histology). This also displays the robustness of our decellularization protocol, which was previously used to decellularize porcine Achilles’ tendon and porcine and human anterior cruciate ligament tissue [5, 37]. Decellularization and sterilization did not influence the mechanical, structural, and compositional properties that all maintained within the range of native meniscus tissue. Also, the in vitro cytotoxicity was not affected by the procedure. Thus, a human meniscus allograft was produced that is proposed to be immune-privileged in a clinical setting due to its acellular nature.

In this research, decellularization and sterilization did not significantly affect biomechanical properties, suggesting that the mechanical behavior of human native meniscus tissue can be maintained. This is promising since previous research reported altered mechanical properties, e.g., a lower Young’s modulus and decreased hysteresis area and peak stress of hysteresis [22]. Moreover, research by Gelse and colleagues showed that the reduced dynamic modulus in their decellularized sheep menisci did not improve during 26 weeks in vivo [12], showing the importance of retained native-like mechanical properties at implantation. It should be noted that the mechanical properties of the whole meniscus have not been tested, which is very challenging to achieve in a standardized manner. Therefore, researchers have often used geometrically standardized biopsies to estimate the mechanical behavior [22, 24, 29, 42], similar to our approach. This enabled us to investigate differences in mechanical properties as a consequence of decellularization in a standardized manner, rather than on the mechanical behavior of the meniscus as a whole that are subject to differenced caused by the methodology. We expect the comparison of mechanical behavior between these biopsies to also be valid for complete meniscus tissue.

Since meniscal biomechanical properties are closely related to the structure and organization of the collagen network and GAG content, the maintained mechanical properties suggest that ECM components were not affected by decellularization and sterilization. Indeed, hydroxyproline content was not significantly altered upon decellularization and sterilization and no damaged collagen fibers were found histologically. This is in agreement with previous research into decellularized porcine and rabbit menisci [17, 34]. Since the collagen’s specific crimp pattern is similarly observed on tissue sections of all experimental groups, it supports the maintained mechanical properties found after mechanical testing.

Maintaining GAGs in decellularized tissue is important, but also experimentally challenging. GAGs increase water content and a loss in GAGs can decrease load-bearing capacity. In this research, no significant loss in GAGs was found upon decellularization and sterilization. Yusof et al. [42] and Monibi et al. [28] however reported a loss in GAGs after decellularization of bovine and canine menisci, both quantitatively and visually using immunohistochemical staining [28, 42]. Additionally, Stapleton et al. reported a quantitative loss in GAGs as well [34]. In comparison to the decellularization protocol used in this research, their protocols make use of Sodium Dodecyl Sulfate (SDS), which has been reported to be responsible for removing GAGs [13]. Nevertheless, others have shown that decellularization of porcine menisci tissue using SDS with GAG preservation is possible [15], which might be explained by differences in tissue origin and age [3].

Detergents have been used in several decellularization protocols and have been used to decellularize numerous tissues, varying from tendon, nerves, dermis, and trachea to heart valves [40]. Two of the main detergents used in the soft musculoskeletal decellularization field are Triton X-100 (non-ionic) and SDS (ionic). On a molecular level, Triton X-100 disrupts DNA-protein, lipid-lipid, and lipid-protein interactions, whilst SDS solubilizes cell and nucleic membranes [3, 30, 33], demonstrating that both can be used to permeabilize cell membranes. They both have their advantages and disadvantages and their efficiency in tissue decellularization varies with tissue type and donor age [3]. It is, therefore, disputable which detergent is favorable to decellularize tissues without affecting the native tissue structure. On top of that, residuals from both detergents are detected in decellularized tissue and are often hard to completely wash out [40], while it is important to effectively remove residuals of these detergents to diminish adverse in vivo side effects [30]. For example, remnants of SDS on acellular muscle-derived matrices have been shown to induce a severe inflammatory response in rats [11]. Therefore, non-detergent-based decellularization would be ideal. Preliminary results on ligamentous tissue within our lab show that a freeze-thawing pretreatment followed by an enzymatic incubation (using Benzonase) results in a percentual dsDNA decrease approximately equal to the Triton X-100-mediated decellularization used in this study. This indicates that mechanical disruption of cell membranes may suffice in efficiently lysing cells to facilitate enzyme-mediated DNA breakdown. Since these results are promising, we are currently further investigating non-detergent-based decellularization options.

In this study, in vitro characterization and validation of the decellularized and sterilized meniscus allograft have been performed. The decellularized human meniscus samples showed a dsDNA removal of around 90% and no visible cells and nuclear material on decellularized and sterilized H&E and DAPI stained tissue sections. Although it is claimed that tissues from which most of the visible cellular material is removed are safe to use for implantation [13], it is valuable to examine the biological consequences of the little left-over nuclear material or cytoplasmic debris. This should be examined in future in vivo experiments. For successful in vivo implantation of the decellularized meniscus allograft, ideally, the allograft would be repopulated with autologous cells that can contribute to tissue maintenance. Since the meniscal ECM has a thick and dense structure, in vivo cell infiltration may be problematic. Gelse and colleagues observed cell repopulation on both the surface and periphery of decellularized menisci, but not in the center of the meniscus after 26 weeks of in vivo implantation [12]. To overcome this, decellularized human menisci were previously provided with needle-punched pores, enabling cells to migrate deeper into the meniscal tissue in vitro [31]. This resulted in DNA content to increase, compared to untreated controls without affecting cell viability or mechanical properties [31].

The major clinical challenge will be to have off-the-shelf meniscus grafts with a geometry that matches the (damaged) native meniscus, to fully restore its function. Proper load transmission is essential for achieving natural tibiofemoral contact pressures in absence of local stress concentrations, in particular at the native tissue – graft interface, which would increase the risk for re-ruptures [7]. Therefore, there would be a need for pre-operative evaluation of knee dimensions using e.g., Computed Tomography, or Magnetic Resonance Imaging in combination with e.g. finite element modeling to study knee mechanics. Integration of the decellularized meniscus using bone blocks would be a possible solution since this allows for proper insertion, size matching, and replacement of affected tissue while maintaining the anatomy. Bone blocks can, however, only be used for medial menisci, as there is a risk of tibial tunnel communication for lateral menisci [34]. Adequate sizing of the allograft tissue will still undoubtedly become a clinical challenge, in addition to the number of available proper donors with non-degenerated tissue, which has also been a limitation of the current study.

## Conclusion

Human meniscus tissue was successfully decellularized, while maintaining biomechanical, structural, and compositional properties. Moreover, processed human meniscus sections showed no signs of in vitro cytotoxicity. Together, these results reveal that a decellularized human meniscus allograft has excellent potential for the development of a tissue-engineered solution for off-the-shelf meniscus replacement.

## Supplementary Information

**Additional file 1.**

## Data Availability

The datasets used and/or analysed during the current study are available from the corresponding author on reasonable request.

## Abbreviations

DAPI: 4′,6-diamidino-2-phenylindole dihydrochloride
dsDNA: double-stranded DNA
ECM: extracellular matrix
GAG: glycosaminoglycans
H&E: hematoxylin & eosin
HYP: hydroxyproline
LDH: lactate dehydrogenase
PBS: phosphate buffered saline
scCO_2_: supercritical carbon dioxide
SDS: sodium dodecyl sulfate
