# Supplementary material for: A decellularized and sterilized human meniscus allograft for off-the-shelf meniscus replacement
Source: J Exp Orthop. 2022 Dec 5;9:116. doi: 10.1186/s40634-022-00555-y (PMC9719875; doi:10.1186/s40634-022-00555-y)
Supplement: Supplementary file 1 — Additional file 1. [file 40634_2022_555_MOESM1_ESM.docx]

**Supplementary information**

**Materials and Methods**

**Determination of hydroxyproline content**

To measure the HYP content, digested samples were centrifuged for 10 minutes at 12000 rpm. 75 μL of sample was hydrolyzed with 25 μL 16M NaOH solution (VWR), after which samples and standards (Hydroxyproline (Sigma; H5534)) were autoclaved for 10 minutes at 120°C. Then, 50 μL and 100 μL 1.4M citric acid (Merck) were mixed with the standards and samples, respectively, and centrifuged for 5 minutes at 12000 rpm. Next, 250 μL Chloramin-T solution (Sigma-Aldrich) was mixed with 25 μL of standard and sample, which were 1:1 diluted with 4M NaOH and 1.4M citric acid and incubated for 20 minutes at room temperature. 250 μL aldehyde/perchloric acid solution [150 mg 4-(Dimethylamino)benzaldehyde (Sigma-Aldrich) per mL N-Propanol (VWR), 70% perchloric acid (Fluka), and 4% MilliQ solution] was added and incubated for 15 minutes at 65°C and 300 rpm. 100 μL of both standards and samples were transferred to a 96-wells plate in duplo and absorbance was measured at 550 nm. The amount of HYP present in the samples was calculated based on the standards and normalized to sample dry weight, obtained before digestion.

**Determination of glycosaminoglycan content**

To measure GAG content, digested samples were centrifuged for 10 minutes at 12000 rpm and diluted with digestion buffer (1:10). Then diluted samples and standards (Chondroitin Sulfate Sodium salt from shark cartilage (Sigma-Aldrich, C4384)) were centrifuged for 5 minutes at 12000 rpm and 40 μL of both samples and standards were transferred to a 96-wells plate in duplo, in which it is was mixed with 150 μL DMMB solution [46µM 1-9-dimethylene blue (Sigma-Aldrich), 40.5mM Glycin (VWR) and 40.5mM NaCl (Merck), pH 3.0]. Absorbance was measured at a wavelength of 540 nm, subtracted by the absorbance measured at 595 nm. The number of GAGs present in the samples was calculated based on the standards and normalized to sample dry weight, obtained before digestion.
